# Suppression of different classes of somatic mutations in *Arabidopsis* by *vir* gene-expressing *Agrobacterium* strains

**DOI:** 10.1186/s12870-015-0595-1

**Published:** 2015-08-26

**Authors:** Jasmine M. Shah, Anantha Maharasi Ramakrishnan, Amit Kumar Singh, Subalakshmi Ramachandran, Unnikrishnan Unniyampurath, Ajitha Jayshankar, Nithya Balasundaram, Shanmuhapreya Dhanapal, Geoff Hyde, Ramamurthy Baskar

**Affiliations:** Department of Biotechnology, Bhupat and Jyoti Mehta School of Biosciences, Indian Institute of Technology-Madras, Chennai, 600036 India; Department of Plant Science, Central University of Kerala, Kasaragod, 671328 India; Department of Environmental Science, Central University of Kerala, Kasaragod, 671328 India; 14 Randwick St, Sydney, 2031 Australia

## Abstract

**Background:**

*Agrobacterium* infection, which is widely used to generate transgenic plants, is often accompanied by T-DNA-linked mutations and transpositions in flowering plants. It is not known if *Agrobacterium* infection also affects the rates of point mutations, somatic homologous recombinations (SHR) and frame-shift mutations (FSM). We examined the effects of *Agrobacterium* infection on five types of somatic mutations using a set of mutation detector lines of *Arabidopsis thaliana*. To verify the effect of secreted factors, we exposed the plants to different *Agrobacterium* strains*,* including wild type (Ach5), its derivatives lacking *vir* genes, oncogenes or T-DNA, and the heat-killed form for 48 h post-infection; also, for a smaller set of strains, we examined the rates of three types of mutations at multiple time-points. The mutation detector lines carried a non-functional β-glucuronidase gene (*GUS*) and a reversion of mutated *GUS* to its functional form resulted in blue spots. Based on the number of blue spots visible in plants grown for a further two weeks, we estimated the mutation frequencies.

**Results:**

For plants co-cultivated for 48 h with *Agrobacterium*, if the strain contained *vir* genes, then the rates of transversions, SHRs and FSMs (measured 2 weeks later) were lower than those of uninfected controls. In contrast, co-cultivation for 48 h with any of the *Agrobacterium* strains raised the transposition rates above control levels. The multiple time-point study showed that in seedlings co-cultivated with wild type Ach5, the reduced rates of transversions and SHRs after 48 h co-cultivation represent an apparent suppression of an earlier short-lived increase in mutation rates (peaking for plants co-cultivated for 3 h). An increase after 3 h co-cultivation was also seen for rates of transversions (but not SHR) in seedlings exposed to the strain lacking *vir* genes, oncogenes and T-DNA. However, the mutation rates in plants co-cultivated for longer times with this strain subsequently dropped below levels seen in uninfected controls, consistent with the results of the single time-point study.

**Conclusions:**

The rates of various classes of mutations that result from *Agrobacterium* infection depend upon the duration of infection and the type of pathogen derived factors (such as Vir proteins, oncoproteins or T-DNA) possessed by the strain. Strains with *vir* genes, including the type used for plant transformation, suppressed selected classes of somatic mutations. Our study also provides evidence of a pathogen that can at least partly counter the induction of mutations in an infected plant.

**Electronic supplementary material:**

The online version of this article (doi:10.1186/s12870-015-0595-1) contains supplementary material, which is available to authorized users.

## Background

*Agrobacterium tumefaciens,* the original and pre-eminent tool for the transfer of genes into plants, has played central roles in both fundamental research and the development of many useful transgenic plants [1–3]. The ready exploitability of *Agrobacterium* for plant transformation derives from its ability, in nature, to transfer a segment (T-DNA) of its own extrachromosomal DNA (the Ti plasmid) into the nuclear DNA of a wide variety of plants [4]. In the laboratory, disarmed strains of *Agrobacterium* can be engineered in which most of the T-DNA segment of the plasmid is replaced with one or more foreign genes of interest, comprising a novel T-DNA. Upon co-cultivation with the target plant, this T-DNA will be transferred, with the involvement of a number of virulence (Vir) proteins encoded on the plasmid’s non-T-DNA portion or on another plasmid in the same bacterium, to the plant’s nuclear DNA [5, 6].

While replacing the native T-DNA of *Agrobacterium* with a novel T-DNA can be done with great precision, the overall process of transformation introduces many unpredictable and unwanted effects on genetic or epigenetic (hereafter, genomic) integrity. The best characterised of these are certain types of somatic mutations (deletions, insertions, translocations) caused by the introduction of the novel T-DNA [7–11]. The T-DNA-induced mutations could be identified as such because of their association with a border region of the T-DNA insert. *Agrobacterium*-mediated transformation of plants with a disarmed strain has also been found to (a) increase the frequency of another class of mutations, i.e. transpositions (in *Arabidopsis*, [12–14]); (b) alter the methylation status of the organism [15]; and (c) cause RNA silencing [15]. It is also possible that *Agrobacterium*-mediated transformation with a disarmed strain has many other as yet unreported impacts on genomic integrity. Natural infections with other plant pathogens, for example, result in higher rates of recombination, double-strand breaks and transposition [16–23]. Even though plants transformed with a disarmed strain of *Agrobacterium* are not induced to grow tumours (the novel T-DNA typically lacks the necessary oncogenes), there are several reasons why the mutational effects of disarmed strains might mimic those of natural infections. The bacterium’s engagement with the plant should be sufficient to induce a generic defence response: It must attach to a cell, breach the wall and plasma membrane, and deliver the T-DNA into the cell [3, 5]. Also, apart from the novel T-DNA, the host plant is exposed to other secretory factors from *Agrobacterium* such as Vir proteins and MAMPs (microbe-associated molecular patterns). Finally, infection is a form of stress for a plant, and given that abiotic stress is known to cause some of the genomic impacts already mentioned, and also some additional types (e.g. increased frequency of point mutations and decreased microsatellite stability) [24, 25], then the stress component of transformation, with a natural or disarmed strain, could also have comparable unintended effects. However, and as already implied, to our knowledge there have been no studies on the possible effects of *Agrobacterium*-mediated transformation (of any type) on the frequency of point mutations (i.e. transitions and transversions), frame-shift mutations (not linked to T-DNA), nor on the frequency of somatic homologous recombinations (SHRs).

The significance of a more robust investigation of transformation goes beyond understanding the unintended changes to the first generation transformants. In *Arabidopsis* for example, increased transposition rates due to *Agrobacterium*-mediated transformation have been found to persist through at least six generations [12]. Pathogen infected plants are known to have progeny with altered genetic (e.g. SHR [16, 17, 26]) and / or epigenetic (e.g. methylation, [17]) imprints. Somatic mutations in general have also been shown to be heritable [27–30].

In this study, we have determined the frequency of five different classes of mutations (transversions, transitions, SHRs; transpositions and frame-shift mutations (a measure of microsatellite instability [31]) in *Arabidopsis* seedlings exposed to four different strains of *A. tumefaciens* (Table 1), *E.coli*, and heat-killed versions of the wild-type strains of *A. tumefaciens* and *E.coli*. The strains of *Agrobacterium* used (Table 1) were chosen because they carry various combinations of three features of the wild type, namely: the virulence (*vir*) genes (V), the oncogenes (O) and a T-DNA (T). One strain had all three features (hereafter, VOT), a second was lacking oncogenes (VXT), a third was lacking both oncogenes and a T-DNA (VXX) and a fourth had none of the three features (XXX). By comparing how these strains (and also *E. coli* and heat-killed bacteria) affect mutational frequencies we could tease out the relative contributions of the generic plant defence response and *Agrobacterium*-specific factors. Our methodology, including the use of two-day old, readily disinfectable seedlings, reduces the influence of several unwanted sources of experimental and biological variability. Unexpectedly, we found that the *Agrobacterium* strain most similar to that used to create transgenic plants suppressed, or did not affect, the rates of mutation in four of the classes of mutation studied. In the three classes of mutation where suppression was observed, the presence of *vir* genes was sufficient for a strain to cause the response. In addition to its relevance to bioengineering, our study also provides evidence of a plant pathogen capable of suppressing somatic mutations in the host plant.

**Table 1:** Different *A. tumefaciens* strains derived from wild type Ach5

*Agrobacterium* strain	Descriptor	Characteristic features	T-DNA	Vir proteins	Oncogenes
Ach5	VOT	Tumor-inducing strain, with Ti plasmid	Yes	Yes	Yes
LBA4002	XXX	Non-tumorigenic strain, without Ti plasmid	No	No	No
LBA4404	VXX	Non-tumorigenic strain, with disarmed Ti plasmid	No	Yes	No
LBA4404 (pCAMBIA2300)	VXT	Non-tumorigenic strain, with disarmed Ti plasmid and a binary vector	Yes	Yes	No

## Results

### Infection with *vir* gene-harbouring *Agrobacterium* strains suppressed rates of transversion, but not transition, in *Arabidopsis*

**Fig. 1:**
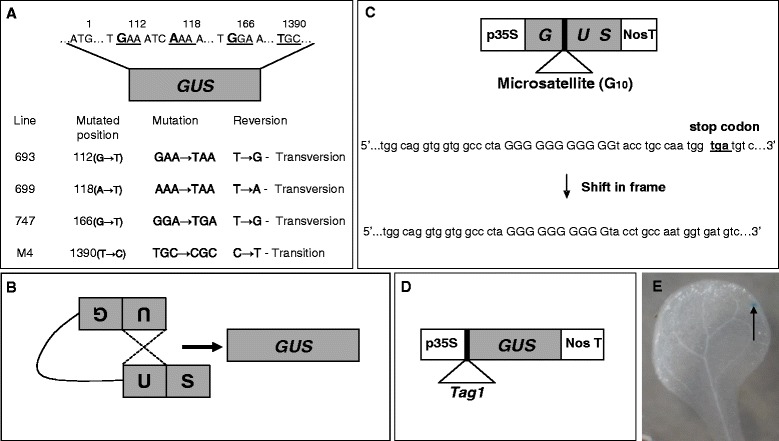
Constructs for scoring somatic mutation rates. **a**. Construct for scoring point mutations, showing the positions and types of mutated bases in the open reading frame of the *GUS* gene, [32, 33]. **b**. Gene cassette with an inverted repeat of the truncated *GUS* gene [34]. **c**. Construct to detect frame-shift mutations [36]. **d**. Construct to score transposition frequency; the *Tag1* element is engineered between the *GUS* and a CaMV 35S promoter [37]. **e**. A blue spot (arrow), showing functional *GUS* reversions after histochemical staining

**Fig. 2:**
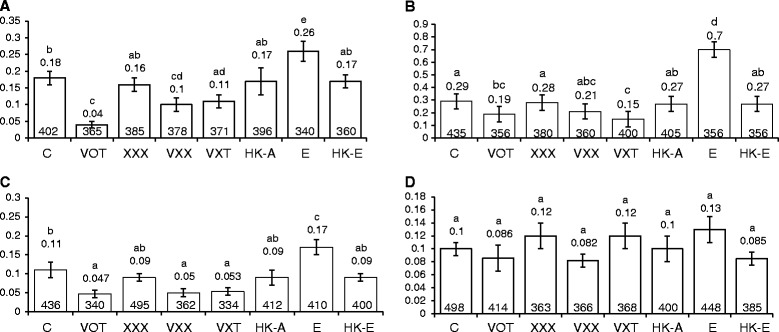
Point mutation rates in different *Arabidopsis* detector lines, after infection with various *Agrobacterium* strains, *E. coli* and heat-killed bacteria for 48 h. **a**. T → G transversion rates in line 693. **b**. T → G transversion rates in line 747. **c**. T → A transversion rates in line 699. **d**. C → T transition rates in line M4. Bars indicate the standard error of the mean of three biological repeats, each consisting of about 140–160 plants. Numbers on top of the bars show the respective mean values. Numbers at the bottom show the respective number of seedlings analysed. Vertical axis shows the mutation rates. ^a, b, c, d, e^Means not followed by the same letter are significantly different at 5 % level as determined by Duncan’s multiple range test. C, control; HK-A, heat-killed-Ach5; E, *E. coli*; HK-E, heat-killed *E. coli*

To examine if infection selectively alters either the spontaneous transition or transversion rates, *Arabidopsis* plants (accession Columbia) carrying different versions of a point mutated *GUS* reporter gene [encoding β-glucuronidase (GUS)] driven by CaMV-35S promoter [32, 33] (Fig. 1a) were used. Transversion rates were analysed using the lines 693, 699 and 747, in which stop codons were introduced in the *GUS* open reading frame (ORF) in three different positions (112_G→T_, 166_G→T_ and 118_A→T_, respectively (Fig. 1a) [32]. Transition rates were examined using the mis-sense *GUS* mutant line M4 (T → C), where base T was mutated to C at the 1390^th^ position in the ORF of *GUS* (Fig. 1a; [33]). Two-day-old seedlings of the transition and transversion detector lines were simultaneously co-cultivated with *Agrobacterium* strains VOT, VXT, VXX and XXX (Table 1), heat-killed VOT, *E. coli* and heat-killed *E. coli*. Infection with XXX had no effect on the transversion rates (*P* > 0.05) in any line when compared to uninfected controls (Fig. 2a, b, c). However, infecting the plants with any of the *vir*-harbouring strains (VOT, VXT, VXX) led to a decrease in transversion frequency in all three lines (Fig. 2a, b, c). When the three detector lines were infected with *E. coli*, transversion rates increased sharply (*P* < 0.05), but heat-killed VOT and *E.coli* had no effect (Fig. 2a, b, c). We observed no significant change in the C → T transition frequency (*P* > 0.05) in plants of the detector line M4 that were infected with either the live *Agrobacterium* strains, live *E. coli*, or the heat-killed strains (Fig. 2d). Thus, *Agrobacterium* strains that harbour *vir* genes selectively suppressed transversions (T → G and T → A), but no strains had any influence on transition (C → T) rates.

### Infection with *vir* gene-harbouring *Agrobacterium* strains reduced the intrachromosomal somatic recombination rates in *Arabidopsis*

**Fig. 3:**
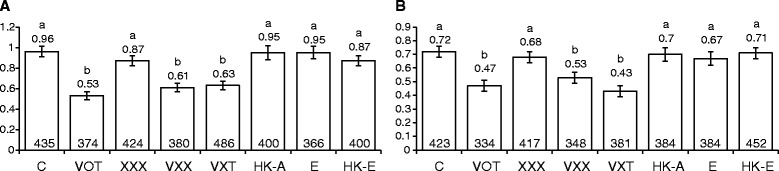
Intrachromosomal somatic homologous recombination rates in *Arabidopsis* detector lines 651 and R2L1, after infection with different *Agrobacterium* strains, *E. coli* and heat-killed bacteria for 48 h. **a**. Line 651 **b**. Line R2L1. Bars indicate the standard error of the mean of three biological repeats, each consisting of about 140–160 plants. Numbers on top of the bars show the respective mean values. Numbers at the bottom show the respective number of seedlings analysed. Vertical axis shows the mutation rates.^a, b^Means not followed by the same letter are significantly different at 5 % level as determined by Duncan’s multiple range test. C, control; HK-A, heat-killed-Ach5; E, *E. coli*; HK-E, heat-killed *E. coli*

Intrachromosomal SHR frequency was scored using *Arabidopsis* lines, 651 (accession C24) and R2L1 (accession Columbia). Line 651 carries an inverted repeat of truncated *GUS* with a sequence homology of 566 bp in the inverted repeat region (Fig. 1b; [34]). A gene encoding for hygromycin resistance is engineered between the two truncated regions. In line R2L1, the *GUS* gene cassette is interrupted by two inverted catalase introns (589 bp) and recombination between the homologous regions restores the functional *GUS* activity [35]. Two-day-old seedlings of lines 651 and R2L1 were co-cultivated with *Agrobacterium* strains VOT, VXT, VXX and XXX (Table 1), heat-killed VOT, *E. coli* and heat-killed *E. coli* for two days. In both detector lines, infection with XXX had no effect on SHR rates (*P* > 0.05; Fig. 3a and b), while infection with any of the *vir* gene-harbouring strains (VOT, VXT, VXX) led to a significant decrease in SHR rates (*P* < 0.05; Fig. 3a and b), compared to uninfected plants. Heat-killed VOT treatment did not affect the SHR rates (*P* > 0.05) in either of the lines, nor did infecting the plants with *E. coli* or its heat-killed form (*P* > 0.05) (Fig. 3a and b). Thus, in addition to transversions, the *vir* genes of *Agrobacterium* seem sufficient to induce the suppression of a second type of mutation, i.e. somatic homologous recombinations.

### Infection with *vir* gene-harbouring *Agrobacterium* strains suppressed frame-shift mutation (FSM) rates in plants

**Fig. 4:**
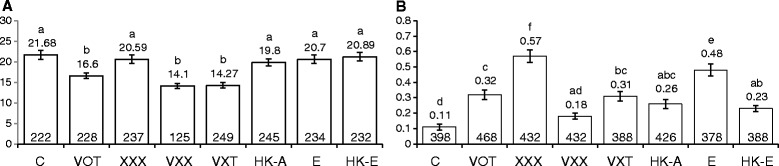
Frame-shift mutation and transposition rates in transgenic *Arabidopsis* detector lines after infection with different *Agrobacterium* strains, *E. coli* and heat-killed bacteria for 48 h. **a**. Frame-shift mutations rates in line G10. **b**. *Tag1* transposition rates. Bars indicate the standard error of the mean of three biological repeats, each consisting of about 60–80 plants. Numbers on top of the bars show the respective mean values. Numbers at the bottom show the respective number of seedlings analysed. Vertical axis shows the mutation rates. ^a, b, c, d, e, f^Means not followed by the same letter are significantly different at 5 % level as determined by Duncan’s multiple range test. C, control; HK-A, heat-killed-Ach5; E, *E. coli*; HK-E, heat-killed *E. coli*

For scoring FSM rates, we used the mutation detector line G10, carrying a microsatellite insertion of 10 Gs within the *GUS* gene ([36]; Fig. 1c). The presence of a stop codon renders *GUS* inactive in G10 plants. Microsatellite regions are prone to errors during replication and hence, addition or deletion of nucleotides during DNA replication in the microsatellite region shifts the reading frame, eliminating the stop codon; a reversion within the *GUS* gene restores the functional form. G10 seedlings were subjected to infection for 2 days (Table 1). Infection with XXX had no effect on frame-shift mutation rates (*P* > 0.05; Fig. 4a), while infection with any of the *vir* gene-harbouring strains (VOT, VXT, VXX) led to a significant decrease (Fig. 4a). Heat-killed VOT treatment had no significant effect on the frame-shift mutation rates (*P* > 0.05), nor did infecting the plants with *E. coli* or its heat-killed form (*P* > 0.05; Fig. 4a). Thus, the pattern of frame-shift responses is identical to that seen with SHRs, adding a third class of mutations for which the *vir* genes of *Agrobacterium* seem sufficient to induce suppression.

### High transposition rates after infection with *Agrobacterium,* its derivatives and *E. coli*

Although *Agrobacterium* infection has previously been shown to increase transpositions, earlier studies were conducted using non-oncogenic strains harbouring T-DNA and *vir* genes [12]. We examined the influence of an oncogenic strain (VOT) and non-oncogenic strains (VXT, VXX, XXX) and also *E. coli* and heat-killed *E.coli* and VOT, on transposition rates. We used a line carrying a *Tag1* element engineered between the CaMV-35S promoter and the *GUS* gene (Fig. 1d; [37]) and transposition of *Tag1* restored the functional GUS activity. Seedlings were infected for 2 days. All strains tested resulted in an increased rate of transpositions (up to five-fold for XXX) compared to the rate in uninfected controls, and all these increases were significant (*P* < 0.05), except in the case of VXX. From highest to lowest mean rates, the sequence of strains was: XXX, *E. coli*, VOT, VXT, heat-killed VOT, heat-killed *E. coli,* VXX (for significant and non-significant differences between these responses, see Fig. 4b). The universal increase in transposition rates represents a very different type of response to that seen with the three other classes of mutations, in which the rate nearly always decreased or remained unchanged (except for the increase in transversion rate caused by live *E .coli*). Infecting the seedlings with *Agrobacterium* strains carrying *vir* genes (VOT, VXX and VXT) induced fewer transpositions than the strain without *vir* genes (XXX). This could represent a less complete form of the apparent suppression of other mutations (transversions, SHR and FSM) by strains with *vir* genes.

### *Agrobacterium* infection-influenced rates of transversion, but not transition, in *Arabidopsis* vary with co-cultivation time

**Fig. 5:**
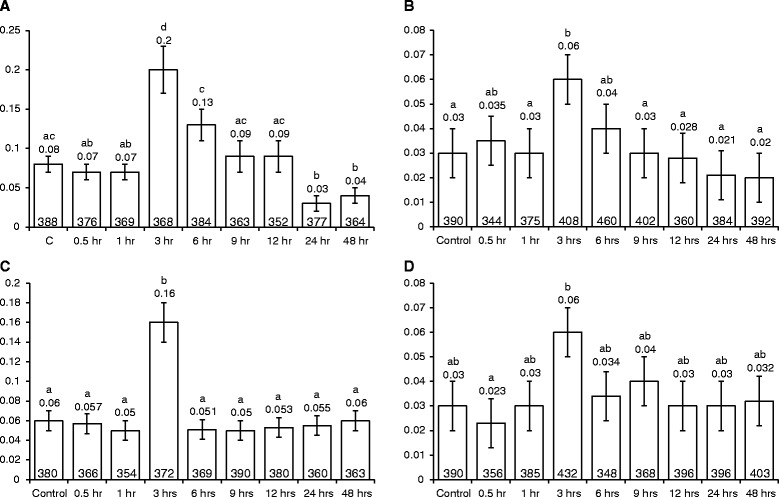
Transversion rates in *Arabidopsis*, after infecting with *Agrobacterium* strain VOT or XXX for various time periods. **a**. T → G transversion rates in line 747 after VOT infection. **b**. T → A transversion rates in line 699 after VOT infection. **c**. T → G transversion rates in line 747 after XXX infection. **d**. T → A transversion rates in line 699 after XXX infection. Bars indicate the standard error of the mean of three biological repeats, each consisting of about 140–160 plants. Numbers on top of the bars show the respective mean values. Vertical axis shows the mutation rates. ^a, b, c, d^Means not followed by the same letter are significantly different at 5 % level as determined by Duncan’s multiple range test

To examine if the rate of transversion depends on the duration of co-cultivation with *Agrobacterium*, we infected the plant lines 747 and 699 (carrying mutated *GUS* meant for analysing (T → G) and (T → A) transversions, respectively) with the two ‘extreme’ *Agrobacterium* strains (VOT and XXX), for different time periods (0.5 h, 1 h, 3 h, 6 h, 9 h, 12 h, 24 h and 48 h). For the infection, two-day old seedlings were used and the GUS staining was done after two weeks of infection. Interestingly, at three hours both strains had caused a sudden and significant increase (of between 2 to 2.7 fold) in transversion rates in both lines (*P* < 0.05; Fig. 5a, b, c, d). This increase had a short-lived nature: by 6 h in all treatments, the rates of transversion had dropped to control levels (Fig. 5a, b, c, d). For plants exposed to strain XXX, the rates remained at control levels for the remainder of the time of the experiments (Fig. 5c, d). For plants exposed to strain VOT, however, the rates continued to show a decreasing trend until 24 h, and in line 747, the levels at 24 h and 48 h were significantly lower than controls by a factor of about 2-fold (*P* < 0.05; Fig. 5a, b). Using a similar experimental design as that described above, we also checked the effects of strains VOT and XXX on (C → T) transition rates in the point mutation detector line M4. We observed no significant change in transition rates with either strain, compared to uninfected controls (see Additional file 1).

These results suggest that in the case of transversions, a factor (or factors) in *Agrobacterium* other than *vir* genes, oncogenes and a T-DNA can cause a short-lived increase in transversion rates at about 3 h, but that one or more of these factors is required for a later dampening of the rate to below control levels.

### *E. coli-*induced rates of transversion, but not transition, vary with co-cultivation time

**Fig. 6:**
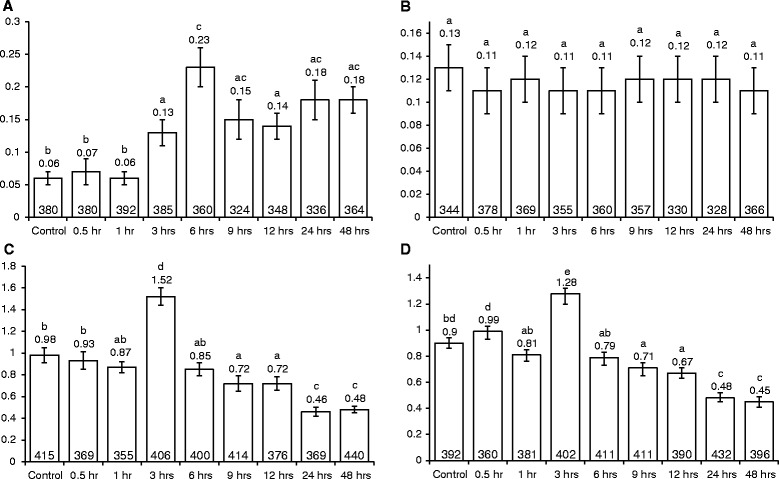
Point mutation and intrachromosomal homologous recombination rates in plants after infection with *E. coli* and VOT respectively, for various time periods. **a**. T → G transversion rates in line 747 after infection with *E. coli*. **b**. C → T transition rates in line M4 after subjecting to *E. coli* treatment **c**. Intrachromosomal homologous recombination rates in line 651 after VOT infection. **d**. Intrachromosomal homologous recombination rates in line R2L1 after VOT infection. Bars indicate the standard error of the mean of three biological repeats, each consisting of about 140–160 plants. Numbers on top of the bars show the respective mean values. Vertical axis shows the mutation rates.^a, b, c, d, e^Means not followed by the same letter are significantly different at 5 % level as determined by Duncan’s multiple range test

The *Arabidopsis* point mutation detector lines, 747 and M4, were subjected to *E. coli* infection for different time periods (0.5 h, 1 h, 6 h, 9 h, 12 h, 24 h and 48 h). T → G transversion rates increased significantly (*P* < 0.05) when line 747 was co-cultivated with *E. coli* for 3 h, 6 h, 9 h, 12 h, 24 h and 48 h (Fig. 6a) and the highest tranversion rates was observed 6 h post infection. However, there was no significant change (*P* > 0.05) in the transition rates when M4 seedlings were co-cultivated with *E. coli* (Fig. 6b).

### VOT-induced somatic homologous recombination (SHR) rates vary with co-cultivation time

To ascertain if SHR rates vary with the duration of co-cultivation with *Agrobacterium*, lines 651 and R2L1 were infected with *Agrobacterium* strains VOT, XXX and *E. coli* independently for periods of up to 48 h. As with the effect of VOT on the transversion rate, the SHR rate also exhibited a short-lived increase in plants exposed to VOT, reaching a maximum at 3 h (*P* < 0.05); the SHR rate then began to fall, dropping below control levels by 9 h, and dropping further again by 24 h (Fig. 6c and d). Unlike the response of transversion rates to XXX, in plants treated with XXX, SHR rates did not change over time, when compared to uninfected controls (see Additional file 2). Likewise, co-cultivation with *E. coli* did not alter the SHR rates throughout the 48 h infection time (see Additional file 3).

## Discussion

Given that transformation mediated by a disarmed strain of *Agrobacterium* is the most common means of producing transgenic plants, it is important to understand as much as possible about the likelihood and nature of any unintended consequences of the approach. We have systematically examined the impact of *Agrobacterium* inoculation on the frequency of five types of spontaneous somatic mutations (Table 2) following the infection of two-day old seedlings of the model plant *Arabidopsis*. Unexpectedly, our results suggest that the predominant effect is an overall suppression of mutations with only one class of mutation (transpositions) exhibiting an increased rate. Interestingly, our results also suggest that when mutations were reduced in frequency this represents the eventual, and *vir*-gene involving, suppression of an earlier, short-lived increase in mutation rate.

**Table 2:** Effect of infection of different *Agrobacterium* strains, *E. coli*, and their heat-killed forms on somatic mutation rates

Strain name		Time interval (hours)	Nature of mutation					
			Transversion		C → T Transition	SHR	FSM	Transposition
			T → G	T → A				
*Agrobacterium*	VOT	0.5	▪	▪	▪	▪	▫	▫
		1	▪	▪	▪	▪	▫	▫
		3	↑↑	↑↑	▪	↑↑	▫	▫
		6	↑	↑	▪	▪	▫	▫
		9	↑	▪	▪	↓↓	▫	▫
		12	↑	▪	▪	↓↓	▫	▫
		24	↓↓	↓	▪	↓↓	▫	▫
		48	↓↓	↓	▪	↓↓	↓↓	↑↑
	XXX	0.5	▪	▪	▪	▪	▫	▫
		1	▪	▪	▪	▪	▫	▫
		3	↑↑	↑	▪	▪	▫	▫
		6	▪	▪	▪	▪	▫	▫
		9	▪	▪	▪	▪	▫	▫
		12	▪	▪	▪	▪	▫	▫
		24	▪	▪	▪	▪	▫	▫
		48	▪	▪	▪	▪	▪	↑↑
	VXX	48	↓	↓↓	▪	↓↓	↓↓	↑↑
	VXT	48	↓↓	↓↓	▪	↓↓	↓↓	↑↑
Heat-killed VOT		48	▪	▪	▪	▪	▪	↑↑
*E. coli*		0.5	▪	▫	▪	▪	▫	▫
		1	▪	▫	▪	▪	▫	▫
		3	↑↑	▫	▪	▪	▫	▫
		6	↑↑	▫	▪	▪	▫	▫
		9	↑↑	▫	▪	▪	▫	▫
		12	↑↑	▫	▪	▪	▫	▫
		24	↑↑	▫	▪	▪	▫	▫
		48	↑↑	↑↑	▪	▪	▪	↑↑
Heat killed *E. coli*		48	▪	▪	▪	▪	▪	↑↑

SHR, somatic homologous recombination; FSM, frame-shift mutation; ▪, no change (*P* > 0.05) compared to uninfected control; ↑↑, significant increase (*P* < 0.05) compared to uninfected control; ↓↓, significant decrease (*P* < 0.05) compared to uninfected control; ↑, increase, but not significant (*P* > 0.05) compared to uninfected control; ↓, decrease, but not significant (*P* > 0.05) compared to uninfected control; ▫, not analysed

When measured 48 h post infection, two-day old *Arabidopsis* seedlings exposed to wild-type *Agrobacterium* (strain VOT) or to a strain with features similar to that used for transformation (VXT) exhibited reduced rates for three classes of mutations: transversions, SHRs and frame-shift mutations (Table 2). This response was not seen in plants exposed to either a heat-killed strain of wild-type *Agrobacterium* or to a live strain (XXX) that lacked three characteristic features of the wild-type: virulence (*vir)* genes, oncogenes and a T-DNA. In both of these cases, the rates for each of these three mutation classes at 48 h post infection were the same as for uninfected controls. Thus, for the reduction in mutation rates, live *Agrobacterium* infection is necessary and it is likely that there was no response to MAMPs from *Agrobacterium*. In an earlier study, MAMPs did not induce DNA double-strand breaks either [23]. With respect to the three characteristic features of the wild-type, the presence of *vir* genes was sufficient. What might the *vir* genes be doing? One possibility is suggested when the results of our time-course experiments are considered in the light of previous studies of generic plant defence responses, and the impacts of infection and stress on the plant genome.

First, a feature of plant infections, or indeed any stressing event, is the initial induction of plant defence genes. For example, when tobacco cells were infected with *Agrobacterium* strains differing in their possession of *vir* genes and a T-DNA, it was shown that for strains carrying both *vir* genes and a T-DNA, the expression of defence genes had increased by 3 – 6 h [38]. Secondly, both infections, or abiotic stressors expected to induce plant defence genes, have been associated with the induction of several classes of mutation (e.g. [20, 21, 23]). Thirdly, plant defence mechanisms help to suppress DNA damage [23]. Interestingly, in our study, where we also studied the time-course of rates of transversions, SHRs and transitions (Table 2), the wild-type strain, VOT, caused a short-lived increase in the first two of these classes, at 3 h, before the rates dropped back to either below or equal to those of uninfected controls. However, the suppression of mutations was observed from 6 h post-infection onwards whereas in tobacco cell culture, the expression of defence genes was highest at 6 h post-infection [38]. This variation could be because of the difference in the model system used. The rates of transitions never differed at any time from those of control plants.

We would like to propose the following model for *Agrobacterium* infection: (1) That an early response of *Arabidopsis* to infection with wild-type *Agrobacterium* (and perhaps with any strain) is the induction of plant defence genes [38, 39]; in this initial stage (about three hours) the plant responds to the infection by increasing the rates of certain somatic mutations (transversions and SHRs; perhaps FSMs) [20, 21] which add to a basal level of uninduced, spontaneous mutations (observed even in the uninfected control). (2) If the plants are infected for six hours or more, then the net effect of the longer exposure is an overall suppression of somatic mutations. This could be because either the stimulatory effect of the shorter exposure does not now occur or the basal level of uninduced, spontaneous mutations is reduced, or both. The suppression of spontaneous mutations could be due, as previously proposed, to certain unknown (and persistent) factors expressed in the host in response to the infection [23]. In interpreting the results, it is important to realize that we were using a pulse-chase design. The infections were relatively brief (mostly 3 h or 6 h) and were carried out on two-day old seedlings which were then disinfected and left for two weeks before the GUS staining was done. In plants infected for 6 h or more, the lower rates of mutation (compared to 3 h plants) might largely represent the surviving ‘imprint’ of the mutations induced by the initial 3 h component of the total period of exposure. While the persistent suppression of spontaneous mutations is sufficient to explain the low rate of mutations in plants exposed to *Agrobacterium* for 6 h or more, it is also possible that a further reduction in the net mutation rates (in plants exposed for 24 h or more) could occur if the infection somehow suppressed the proliferation of any previously induced mutant cells during the two weeks of growth prior to measurement.

Regardless of the specific mechanism/s responsible for the eventual suppression of the overall mutation rate below that seen in controls, it is important to note that this reduction is likely with the types of disarmed strains used for bioengineering. The fully virulent strain (VOT) caused a much greater suppression than the avirulent (XXX) strain; in the latter case, prolonged infection resulted in lower overall rates than those seen with 3 h exposures, but the rates never dropped below control levels.

Several steps of our model have previously been suggested as occurring during infections of *Arabidopsis*, where *Tobacco mosaic virus* and *Oilseed rape mosaic virus* were associated with an induction of plant defence genes and SHRs [20]. The induction of SHRs by *Tobacco mosaic virus* and *Oilseed rape mosaic virus*, for example, is a long-lasting, unsuppressed increase, and even persisted into subsequent generations [17, 20]; in our study, the induction of transversions by live *E. coli* also persisted to the end of the 48 h testing period, perhaps a result of induced plant defence genes (e.g. *PR1* [39])*.* Infection studies of *Arabidopsis* with *Pseudomonas syringae* pv. revealed that the plant’s defence mechanisms do help to suppress DNA damage [23]. The suppression of the spontaneous mutations is to a lesser extent in plants subjected to *E. coli*, when compared to the XXX and that could be because of the differential response of the host towards either of the pathogens. It is also plausible that some of the mutations that provide benefits to a plant [24] could be disadvantageous in the long-term to a pathogen like *Agrobacterium*, and strains with Vir proteins might gain an advantage by the suppression of spontaneous mutations. Our study provides evidence of a pathogen that might be able to counter the infected plant’s mutation-inducing strategy. *Agrobacterium* is one of the most sophisticated of plant pathogens, and a mutation-suppressing capability might represent another powerful tool in its arsenal.

None of strains of *Agrobacterium* or *E. coli* tested had any effect on transition rates, at any time-point (Table 2). One possible reason could be due to low methylation levels in the CpG dinucleotide of the seedlings at the time of *Agrobacterium* and *E. coli* infection. Methylated cytosines are more prone to deamination in the genome [40] and the level of methylation increases throughout *Arabidopsis* development (from cotyledons to vegetative tissues) [41]. The infections were carried out on two-day old seedlings which may not have sufficient levels of methylated CpG to show a significant change in C → T transitions.

Transpositions were the one class of mutation in which the rates in infected plants were higher at 48 h than in control plants. This was true for all strains of *Agrobacterium* or *E. coli* tested, including heat-killed strains of both bacteria; there were however highly significant differences in the magnitude of the increase across the strains. In the absence of time-course data, it is difficult to properly interpret the pattern of observed changes. The net increase in transposition by any strain could, for example, depend on the balance between the strength of an early inducing signal for increased transpositions (e.g. induced plant defence genes [20]) and the strength of a later transposition-suppressing signal (e.g. *vir* genes, as proposed above for other mutational classes). A potential (but in this case, incomplete) transposition-suppressing role for *vir* genes is consistent with our results, since once we exclude the heat-killed strains (which our evidence suggests are unlikely to be strong inducers of any class of mutation), then the strains associated with the lowest rates of transposition are the three that possess *vir* genes. Conversely, the strain associated with the highest rate of transpositions is the *vir* gene-lacking, live, *Agrobacterium* strain, XXX. As to why *Agrobacterium* would be unable to properly suppress the induction of transpositions, this might be due to an unusually strong induction of this class of mutation by bacterial pathogens, and/or a less effective suppression mechanism, compared to those that apparently counter the other mutation types. The strains with *vir* genes (VOT, VXX and VXT) did not differ in their ability to influence point mutations, SHRs, FSM. However, transpositions did seem to increase due to the presence of T-DNA (in strains VOT and VXT), and oncogenes did not seem to contribute to higher transposition rates.

It should be noted that our methodology has been fine-tuned to reduce unwanted variability from several possible sources. By using two-day old seedlings for the initial infection we could analyse the impact of infection on the whole plant and not just on any particular organ. For example, it has previously been reported that recombination rates were higher in *Arabidopsis* leaves than in flowers and stem [42]. Also, all our measurements were made when the plants were at the same age. Previously, it has been found that the rates of microsatellite instability increased [43] and SHR decreased [44] with age in *Arabidopsis* plants. The small size of the seedlings also allows us to manipulate and infect the plants without wounding them [45], which is almost inevitable when handling adult plants. Wounding would induce host defence responses the effects of which we would be unable to distinguish from those due to the infection alone. Wounded plants would also preferentially enhance the expression of *vir* genes in the strains that harbour the *vir* genes (VOT, VXT, VXX) compared to the *vir* gene-lacking XXX strain. Enhanced expression of Vir proteins favours bacterial attachment, a property that the strain XXX lacks. The small size of the plants also allowed us to readily disinfect the plants at times of our choosing post-infection. By leaving the disinfected plants to grow for a further two weeks our mutation counts were more reliable: instead of needing to detect single blue cells (a consequence of the modified *GUS* gene) the mutant foci were readily recognisable blue spots. Ulker et al. (2012) [46] did raise a concern over the reliability of mutation reporter lines and sequence of reporter lines after *GUS* restoration, which was addressed by Puchta and Hohn (2012) [47]. There are several papers published using these lines to score somatic mutations in different circumstances [48, 49]. Further, several other groups have generated a number of transgenic *Arabidopsis* lines to score reversions adopting the same approach [33, 35, 50]. Thus, the approach to identify somatic reversions is robust and well established now. Kovalcuk et al. (2000) [32] have isolated the DNA from those blue spots and have ascertained the reversion of the original mutations and so it is indeed possible to confirm reversions. We subjected the control seedlings to the same conditions as that of the infected seedlings, except that the bacteria were not present in the medium. 400–500 plants were screened for each treatment, which was much more than previous recommendations (~30-100 plantlets per treatment – Puchta and Hohn, 2012) [47]. The mutation frequencies we observed were very low (e.g. between 0.03 to 0.5 events per plant upon VOT infection) and hence screening a large population was necessary.

## Conclusions

1. Infection of *Arabidopsis* seedlings with either the wild-type strain of *Agrobacterium* or a disarmed strain with characteristics similar to that used for bioengineering led (after 48 h of infection) to the suppression of, or had no effect on, four of the five types of mutations measured [transversions, SHRs, FSM (suppressed) and transition (unchanged)].

2. Only the frequency of transpositions was increased, with all strains of *Agrobacterium* tested, and the magnitude of the increase varied greatly across the strains tested.

3. The presence of *vir* genes in the *Agrobacterium* strains used appears sufficient to lead to the suppression of mutations (in those classes where it occurred). No suppression (below the rates observed in uninfected control) was observed when plants were infected with the strain lacking *vir* genes, oncogenes and T-DNA.

4. For transversions and SHRs, the suppression observed in plants infected for 48 h with the wild-type strain represented a reversal of an earlier short-lived increase in frequency, and this reversal was not observed when plants were infected with the strain lacking *vir* genes, oncogenes and a T-DNA.

5. Overall, the study indicates that bioengineering based on transformation with *Agrobacterium* might not necessarily lead to greater rates of somatic mutations, except for the T-DNA-linked mutations and transpositions. Our study also provides the first evidence of a pathogen that can at least partly counter the induction of mutations by an infected plant, a further indication of *Agrobacterium*’s sophisticated infective capabilities.

## Methods

### *Arabidopsis* transgenic lines

Point mutation frequencies were assayed using the transgenic *A. thaliana* (accession Columbia) lines 693, 699, 747 and M4. In lines 693, 699 and 747, stop codons were introduced in the *GUS* ORF at three different positions 112_G→T_, 166_G→T_ and 118_A→T_, respectively (Fig. 1a); these transgenic lines were provided by Igor Kovalchuk (University of Lethbridge, Canada) [32]. Line M4 was provided by Anna Depicker (Ghent University, Belgium) [33]. In line M4, a mis-sense mutant of the *GUS* gene is inserted where the base T is mutated to C at the 1390^th^ position (Fig. 1a). Somatic homologous recombinations were scored using lines 651 and R2L1, where the recombination substrates are inverted repeats of a truncated *GUS* gene (Fig. 1b). Line 651 (C24 ecotype) was gifted by Barbara Hohn (Friedrich Miescher Institute, Switzerland) [34]. We obtained the line R2L1 (Columbia ecotype) [35], as well as the line G10 (Columbia ecotype) [36] from Francois Belzile (University of Laval, Canada). Line G10 has a microsatellite (stretch of 10 Gs) within the *GUS* ORF (Fig. 1c). The *Tag1* line was provided by Nigel Crawford (University of California, California) [37]. All the experiments were conducted on homozygous seedlings.

### Bacterial strains

Infections were carried out using wild type *A. tumefaciens* strain Ach5 (referred to as VOT in the main body of the paper), and its derivatives (Table 1), as well as with *E. coli*. The *Agrobacterium* strains Ach5 and LBA4404 (referred to as VXX in the main body of the paper) were provided by K. Veluthambi (Madurai Kamaraj University, India) [51]. LBA4404 is a derivative of Ach5 with disarmed Ti plasmid pAL4404 and hence, it has only the *vir* and *ori* regions of the Ti plasmid, but not the oncogenes and T-DNA [52]. We generated LBA4404 (pCAMBIA2300) (referred to as VXT) by electroporating LBA4404 with the binary vector pCAMBIA2300. We obtained the strain LBA4002 (referred to as XXX) from Paul J. Hooykaas (Leiden University, the Netherlands). LBA4002 is an Ach5-derivative without the Ti plasmid [53]. All the *Agrobacterium* strains were grown on Luria-Bertani (LB) media at 28 °C. The strain LBA4404 (pCAMBIA2300) was grown on media containing 100 mg/l kanamycin. *E. coli* (strain, DH5α) was grown in LB media at 37 °C. Heat-killed bacteria were obtained by heating the culture at 70 °C for 30 min [39] and the heat killed forms were verified by plating them again in appropriate media. Prior to infection, the bacteria were cultured in liquid LB media with appropriate antibiotics and the suspension (0.6 to 0.9 optical density at 600 nm) was centrifuged at 1,100 X g for 10 min and washed with equal volume of liquid germination media thrice to eliminate traces of LB.

### Plant growth conditions and method of infection

*Arabidopsis* seeds were surface sterilised with 500 μl of 70 % ethanol, rinsed with sterile water and treated with 0.5 % bleach for two minutes. Subsequently, the seeds were washed four times with sterile water. Infection of *Arabidopsis* seedlings was performed according to the protocol of Li et al*.* [54] with minimal modification. The seeds were plated on germination media (sterile Murashige and Skoog (MS) media with 3 % sucrose, pH 5.7). Seed germination was synchronised by keeping the MS plates with seeds in the dark, at 4 °C for 48 h. Then the plates were moved to a growth chamber (Percival, USA), having a uniform light intensity of 8000 lux units under a 16-h light/8-h dark cycle. The temperature was maintained at 22 °C throughout the experiments and the humidity was set at 80 %. After two days, the seedlings were rinsed in liquid germination media containing the bacteria and co-cultivated on germination media for the appropriate time period (0.5 h to 48 h). The control seedlings were treated similarly to the infected ones, except that the liquid medium was devoid of bacteria. The infected as well as control seedlings were then surface sterilised with liquid MS media containing 250 mg/l Cefotaxime and 0.05 % plant preservative mixture (Biogenuix Medsystem Pvt. Ltd., New Delhi, India). These seedlings were gently dropped onto germination media containing 250 mg/l Cefotaxime and 0.05 % plant preservative mixture using a wide-mouthed pipette. Uniform spacing between the seedlings was maintained for all the experiments. These plates were kept in the plant growth chamber in the same conditions mentioned above for two weeks and then used for GUS histochemical staining.

### β-Glucuronidase (GUS) histochemical staining

GUS histochemical staining was performed according to the protocol of Jefferson [55]. The staining buffer (100 mM sodium phosphate buffer [pH 7.0]) contained 1 mM 5-bromo-4-chloro-3-indolyl glucuronide (X-Gluc) (Biosynth, Switzerland), 0.1 % Triton X-100 and 50 μg/ml kanamycin. GUS staining solution (10 ml) was added to six-well plates containing approximately 50 plants per well. These plates were vacuum-infiltrated for 10 min and incubated at 37 °C for 48 h. Subsequently these plants were bleached with 70 % ethanol. The blue spots (Fig. 1e) reflecting mutation reversions were counted using a light microscope (Leica KL300).

### Estimating mutation frequencies

Each experiment was done in triplicate and in total about 400–500 plants were taken for each treatment. For studies involving FSM, about 200–250 plants were taken. Mutation frequencies were expressed as the average number of spots observed per plant [33]. The mutation frequencies of the infected plants and the spontaneous mutation frequencies of the control plants were compared. The mutation frequency data sets were tested for normality [56] and equality of variances [57, 58], and were further subjected to one way Analysis of Variance (ANOVA, α = 0.05) to determine significant effects (*P* < 0.05), if any. Duncan´s multiple range test [58–60] was employed when ANOVA revealed significant differences (*P* < 0.05). All the statistical analyses were performed using STATISTICA version 8 software (Stat Soft Inc.). The data sets were plotted in MS Office - Excel software (Microsoft Inc.).

## Additional files

Additional file 1: Figure S1. C → T transition rates in transgenic *Arabidopsis* line M4, after infection with different *Agrobacterium* strains for various time periods. A. After VOT infection. B. After XXX infection. Bars indicate the standard error of the mean of three biological repeats, each consisting of about 140-160 plants. Numbers on top of the bars show the respective mean values, not significantly different (*P* > 0.05), as analysed by Duncan’s multiple range test. Vertical axis shows the mutation rates. (PPT 125 kb) Additional file 2: Figure S2. Intrachromosomal somatic homologous recombination rates in transgenic *Arabidopsis* lines (lines 651 and R2L1) after XXX infection for various time periods. A. Line 651 B. Line R2L1. Bars indicate the standard error of the mean of three biological repeats, each consisting of about 140–160 plants. Numbers on top of the bars show the respective mean values, not significantly different (*P* > 0.05), as analysed by Duncan’s multiple range test. Vertical axis shows the mutation rates. (PPT 127 kb) Additional file 3: Figure S3. Intrachromosomal somatic homologous recombination rates in transgenic *Arabidopsis* lines after infection with *E. coli* for various time periods. A. Line 651 B. Line R2L1. Bars indicate the standard error of the mean of three biological repeats, each consisting of about 140–160 plants. Numbers on top of the bars show the respective mean values, not significantly different (*P* > 0.05), as analysed by Duncan’s multiple range test. Vertical axis shows the mutation rates. (PPT 128 kb)

## Abbreviations

SHR: Somatic homologous recombination
FSM: Frame-shift mutation
GUS: β-Glucuronidase gene
h: Hour
MS: Murashige and Skoog
MAMP: Microbe-associated molecular patterns
LB: Luria-Bertani
