# Supplementary figures and images for: Suppression of different classes of somatic mutations in Arabidopsis by vir gene-expressing Agrobacterium strains
Source: BMC Plant Biol. 2015 Aug 26;15:210. doi: 10.1186/s12870-015-0595-1 (PMC4549908; doi:10.1186/s12870-015-0595-1)

## Slide 1
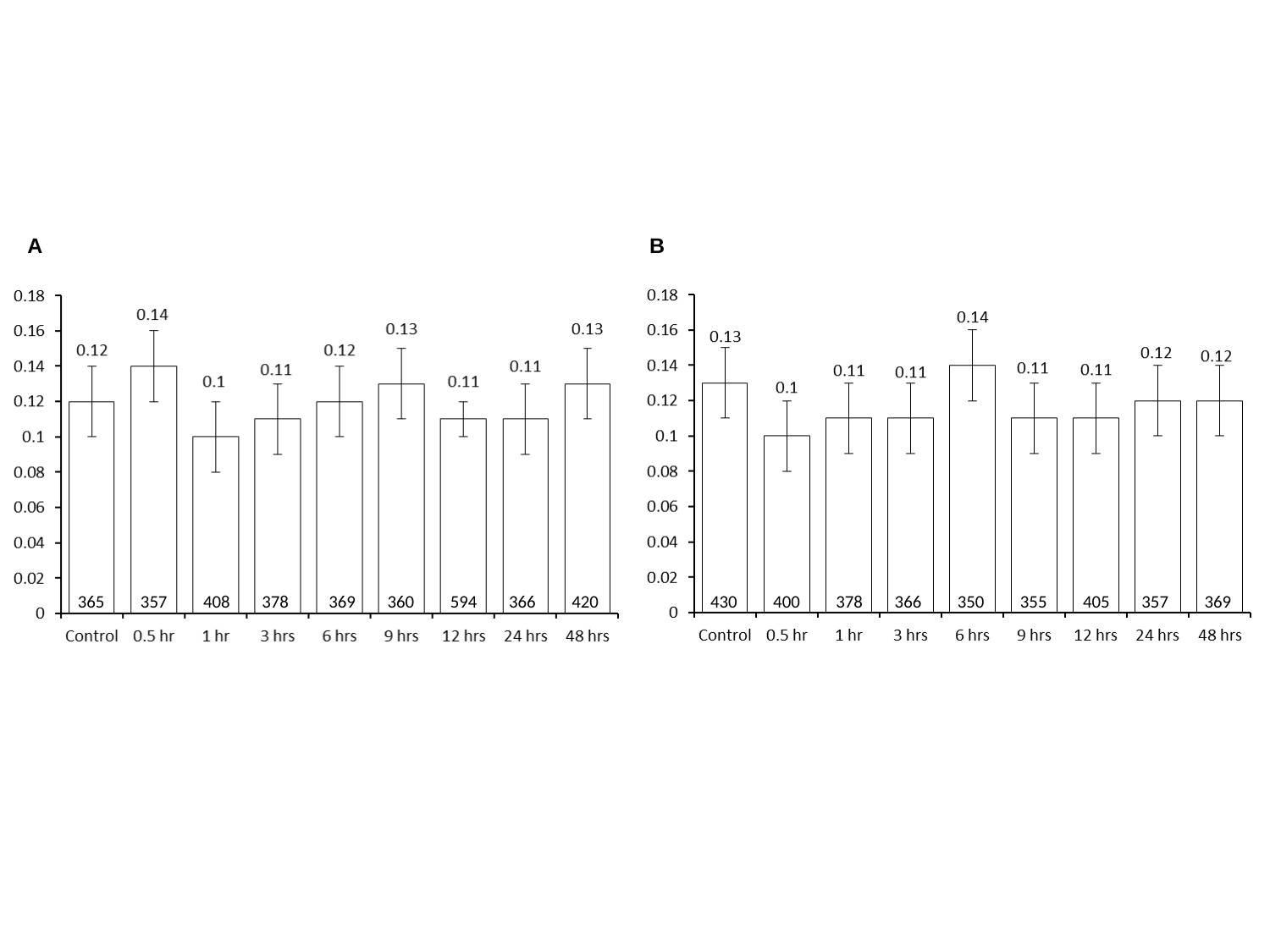

A
B
365 357 408 378 369 360 594 366 420
430 400 378 366 350 355 405 357 369

Supplement: Additional file 1: Figure S1. — C → T transition rates in transgenic Arabidopsis line M4, after infection with different Agrobacterium strains for various time periods. A. After VOT infection. B. After XXX infection. Bars indicate the standard error of the mean of three biological repeats, each consisting of about 140-160 plants. Numbers on top of the bars show the respective mean values, not significantly different (P > 0.05), as analysed by Duncan’s multiple range test. Vertical axis shows the mutation rates. (PPT 125 kb) [file 12870_2015_595_MOESM1_ESM.ppt]

## Slide 1
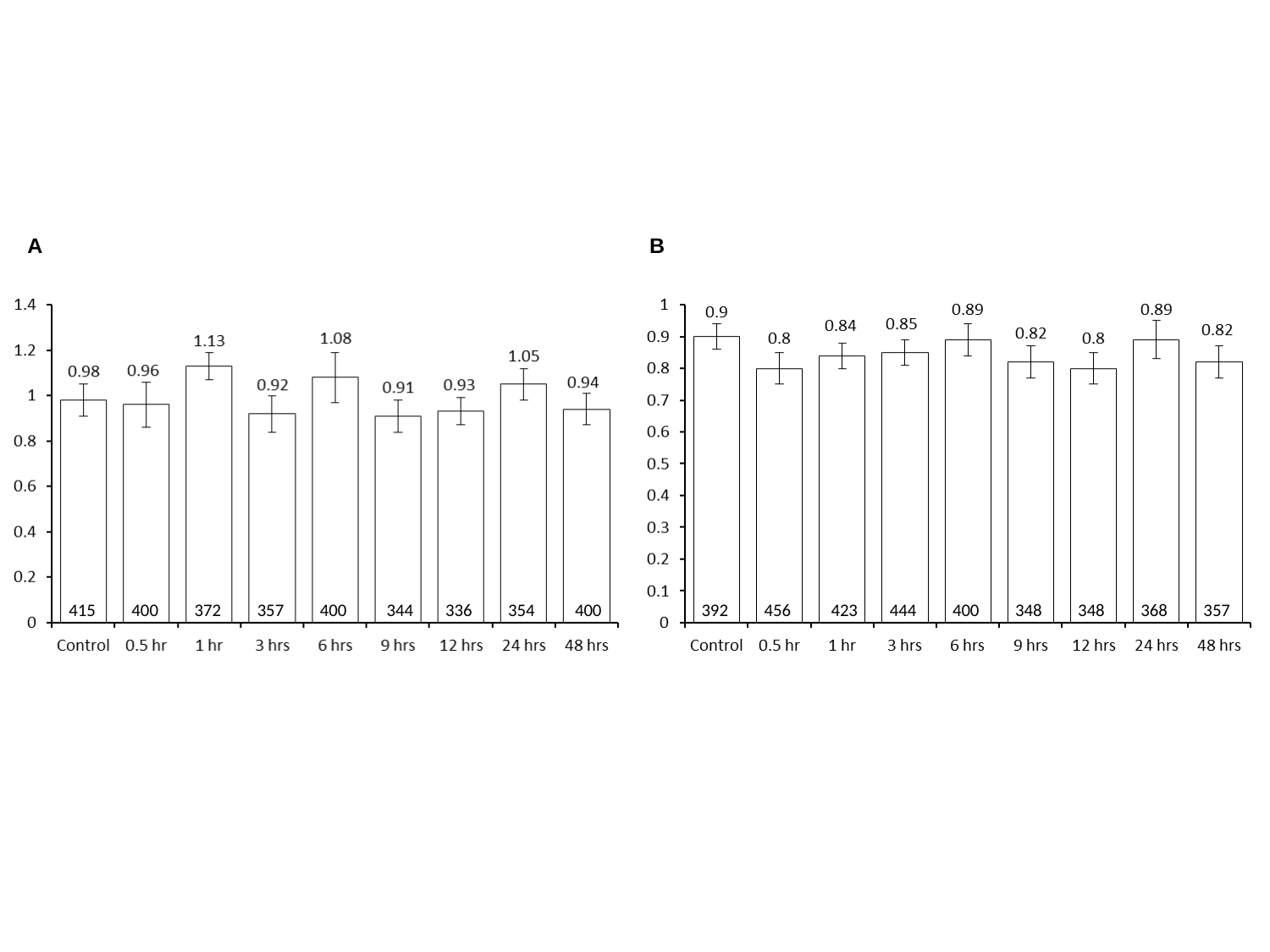

A
B
415 400 372 357 400 344 336 354 400
392 456 423 444 400 348 348 368 357

Supplement: Additional file 2: Figure S2. — Intrachromosomal somatic homologous recombination rates in transgenic Arabidopsis lines (lines 651 and R2L1) after XXX infection for various time periods. A. Line 651 B. Line R2L1. Bars indicate the standard error of the mean of three biological repeats, each consisting of about 140–160 plants. Numbers on top of the bars show the respective mean values, not significantly different (P > 0.05), as analysed by Duncan’s multiple range test. Vertical axis shows the mutation rates. (PPT 127 kb) [file 12870_2015_595_MOESM2_ESM.ppt]

## Slide 1
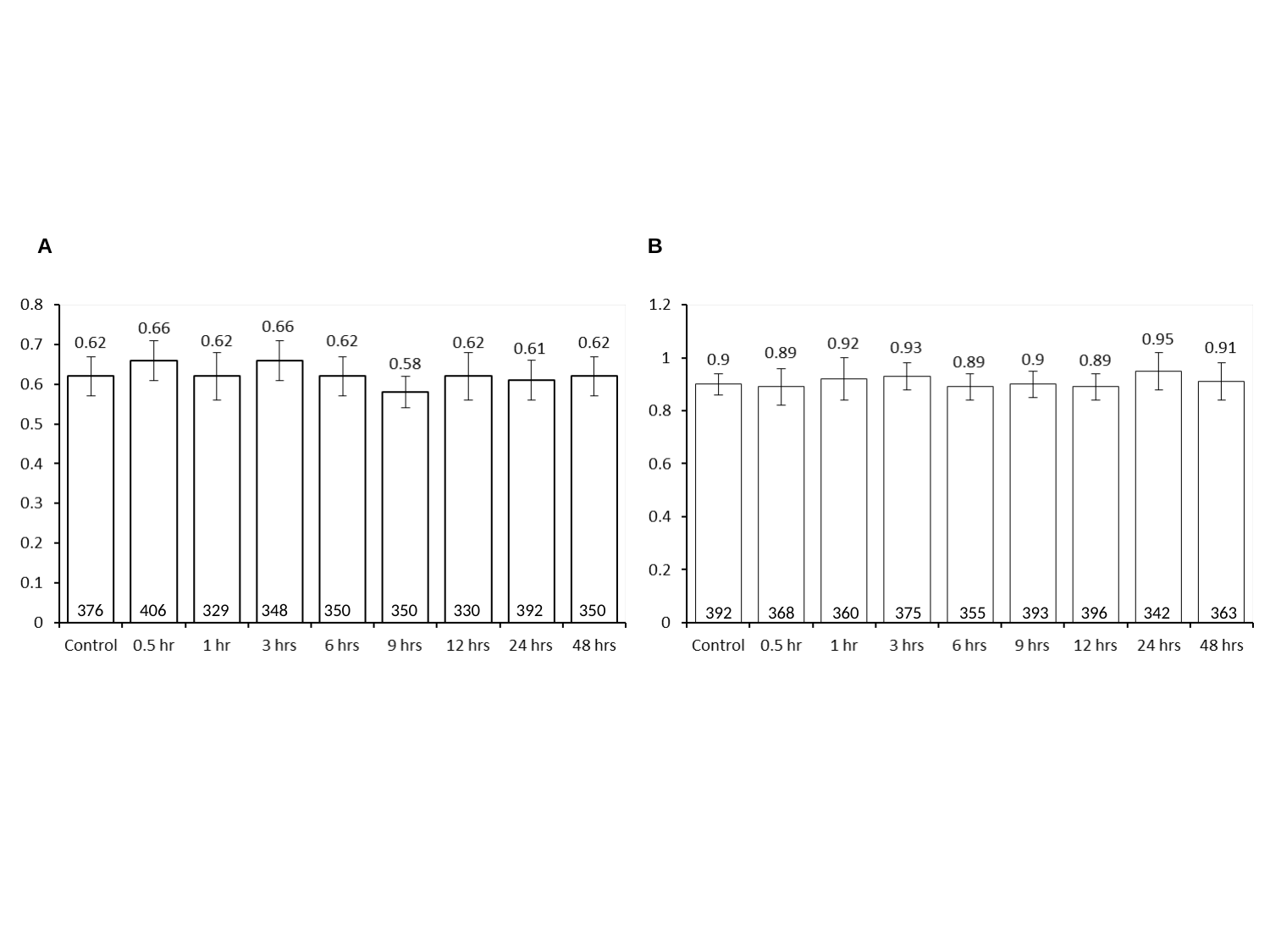

A
B
392 368	360 375	355 393 396 342 363
376 406 329 348 350 350 330 392 350

Supplement: Additional file 3: Figure S3. — Intrachromosomal somatic homologous recombination rates in transgenic Arabidopsis lines after infection with E. coli for various time periods. A. Line 651 B. Line R2L1. Bars indicate the standard error of the mean of three biological repeats, each consisting of about 140–160 plants. Numbers on top of the bars show the respective mean values, not significantly different (P > 0.05), as analysed by Duncan’s multiple range test. Vertical axis shows the mutation rates. (PPT 128 kb) [file 12870_2015_595_MOESM3_ESM.ppt]
